# Current research frontiers in plant epigenetics: an introduction to a Virtual Issue

**DOI:** 10.1111/nph.16493

**Published:** 2020-03-17

**Authors:** Mimmi C. Eriksson, Aglaia Szukala, Bin Tian, Ovidiu Paun

**Affiliations:** ^1^ Botany and Biodiversity Research University of Vienna Rennweg 14 A‐1030 Vienna Austria; ^2^ Vienna Graduate School of Population Genetics Veterinärplatz 1 A‐1210 Vienna Austria; ^3^ Southwest Forestry University Kunming 650224 China

**Keywords:** abiotic stress, biotic interactions, DNA methylation, histone modifications, hybridization, plant development, whole genome doubling

## Abstract

http://www.newphytologist.com/virtualissues

Without directly altering the underlying DNA sequence, epigenetic signals such as histone modifications, DNA methylation and RNA interference (RNAi) can be specific to particular internal or external conditions and they can have phenotypic implications (e.g. Alonso *et al.*, 2019b; Ding *et al.*, 2019; Gehring, 2019; Li *et al.*, 2019; Zhao *et al.*, 2019). Epigenetics was first addressed in *New Phytologist* in 2003 in the context of genomic changes after whole genome doubling (Soltis *et al.*, 2003). A dedicated review on plant evolutionary epigenetics followed in 2005 (Rapp & Wendel, 2005). Since then, *New Phytologist* has published over 80 articles that mention epigenetics, mirroring the steady increase of literature that discusses the biological implications of epigenetic variation (Fig. 1). Following the successful 40^th^ New Phytologist Symposium on ‘Plant epigenetics: from mechanisms to ecological relevance’ (see Heer *et al.*, 2018), this Virtual Issue on the topic is now being released. In this introductory Commentary we highlight several of the included papers that cover four main research frontiers.

**Fig. 1:**
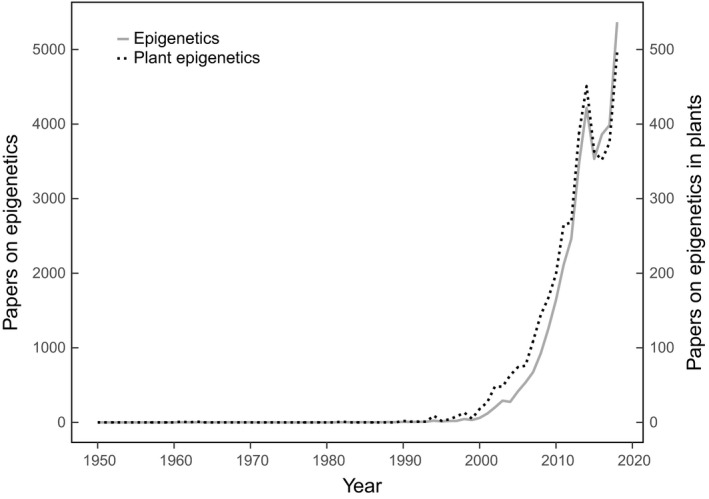
Number of published studies mentioning ‘epigenetics’ (filled line, left scale), and ‘plant’ and ‘epigenetics’ (dotted line, right scale) by publication year during the period 1950–2018 as output by searches in all Web of Science databases (at 15 July 2019).

## Epigenetic regulation of plant responses to abiotic conditions

Epigenetic diversity in natural populations is generally found to be structured and related to environmental variation (Richards *et al.*, 2017), as for example in a large‐scale survey of DNA methylation across populations of *Plantago lanceolata* included in this Virtual Issue (Gáspár *et al.*, 2019). Although a considerable portion of epigenetic variation correlates with trans‐acting genetic variants (Dubin *et al*., 2016; Kawakatsu *et al*., 2016), its nonrandom spatial distribution often exceeds the genetic structuring of natural populations. In addition, transcriptional rewiring through various epigenetic signals has been observed in plants upon multi‐generational exposure to abiotic stresses (Lämke & Bäurle, 2017), such as extreme temperatures (Ding *et al*, 2019; Friedrich *et al.*, 2019), drought (Huang *et al.*, 2019) and salinity (Yang & Guo, 2018). However, a dynamic interplay between specific genetic and epigenetic variants is commonly unravelled by studies of plant stress responses. For example, a loss‐of‐function genetic mutation at JMJ17 histone demethylation in *Arabidopsis thaliana* has been reported to cause a genome‐wide increase in histone 3 lysine 4 trimethylation (H3K4me3) that, in turn, activates multiple dehydration stress‐responsive genes (Huang *et al.*, 2019).

‘Epigenetic diversity in natural populations is generally found to be structured and related to environmental variation …’

Plants that are convergently adapted to extreme abiotic conditions can be particularly useful in understanding the molecular mechanisms that shape these adaptations. Several clades of mangroves, for example, evolved extreme ultraviolet (UV) light‐, salinity‐ and hypoxia‐resistance when independently adapting to the specific intertidal environments. However, such stressful conditions can activate transposable elements (TEs; Lyu *et al.*, 2017). Recently, Wang *et al. *(2018) were able to show that stress‐induced reactivations of TEs become epigenetically controlled by siRNAs‐mediated CHH methylation in the mangrove *Rhizophora apiculata*, but the short active windows of TEs can trigger genetic variation, possibly facilitating adaptation to new conditions.

It is of interest whether epigenetic responses to abiotic stress are localized in the genome, or if they are rather diffuse and multilayered. In a survey across three Brassicaceae species with different levels of resistance to drought, Marín‐de la Rosa *et al*. (2019) observed an overall signal of upregulation of epigenetic reprogramming transcripts in the most drought‐adapted species. They conclude that response to drought is a complex phenotype resulting from an interplay of different traits, and therefore complex underlying regulatory patterns should be expected. Multilayered pathways to modulate gene expression have been indeed reported by Ding *et al*. (2019), this time for plants under cold stress, including regulation by post‐translational histone modifications and DNA methylation, but also by miRNAs and cold‐responsive long noncoding RNAs. A study focused on histone modifications in *Brachypodium distachyon* (Huan *et al.*, 2018) reported vernalization‐induced, dose‐dependent epigenetic changes for multiple genes that coordinate various biological processes to prepare for floral transition. Importantly, the study also reports a quantitative, but rather short‐term epigenetic memory, allowing for a faster response to seasonal temperature changes in the descendant generation.

## Biotic interactions and plant epigenetics

Although highly context‐dependent, plant biotic responses seem to reflect to a certain degree common pathways and epigenetic alterations to those induced by abiotic stress, with or without involving mediating signalling molecules such as jasmonic acid, salicylic acid and reactive oxygen species (Alonso *et al.*, 2019b; Balao *et al.*, 2018; Ding *et al.*, 2019). In this Virtual Issue, Alonso *et al*. (2019b) review the latest knowledge on the epigenetic relevance of biotic interactions, but point out that relevant primary studies are still sparse to allow generalizations. Moreover, most available studies address only the epigenetic consequences of biotic responses, rather than directly approaching the role of epigenetic configurations in determining those responses.

Much of the currently available studies aim to understand plant–pathogen interactions, in particular studying noncoding RNAs. For example, biotrophic fungi infection of *Aegilops tauschii* is reported to trigger a significant downregulation of ARGONAUTE4a that in turn reduces the level of 24‐nt siRNAs and CHH methylation especially for genes near TEs, some with stress response relevant functions (Geng *et al*., 2019). This is, in fact, a more general pattern: epigenetic alterations following biotic stress are frequently observed around genomic regions containing defence‐related genes and their transcriptional activation upon stress is often mediated via neighbouring TEs (Alonso *et al.*, 2019b). In another study (Wang *et al*., 2017), chromatin states driven by both repressive and active histone marks, and facilitated by the presence/absence of a TE upstream of a CCT domain‐containing gene (*ZmCCT*), can lead to resistance to *Gibberella* stalk rot in maize. Additional relevant case studies of plant–animal, plant–microbe and plant–plant interactions are critically discussed in Alonso *et al. *(2019b).

Recent papers (for example Alonso *et al.*, 2019b; Ding *et al.*, 2019; Gáspár *et al.*, 2019; Geng *et al*., 2019) suggest that a better understanding of the epigenetic responses to environmental (i.e. both abiotic and biotic) stress is key to understanding rapid plant adaptation, plant immunity and for developing sustainable strategies for crops’ improvement in the face of global warming. Indeed, the spontaneous and fluctuating nature of epimutations was suggested to enhance the adaptation potential to varying abiotic stimuli (e.g. Becker *et al.*, 2011; Johannes & Schmitz, 2019). Particularly relevant to this, Johannes & Schmitz (2019) review the biological implications of stochastic epimutations, also addressing cases where these lead to trans‐generational changes in gene expression. Nonetheless, epigenetic responses to abiotic stresses that are independent of genetic variation tend, in general, to be transitory after removing the stressor (Gutzat & Mittelsten Scheid, 2012), whereas for biotic stress some true transgenerational effects (i.e. those that are heritable for at least two generations, Lämke & Bäurle, 2017) are visible, but more studies are required to allow for generalizations.

## Epigenetic relevance of hybridization and whole genome doubling

Hybridization and whole genome doubling, individually or in combination (i.e. allopolyploidization), are prevalent in plants and, as genomic stressors, can trigger systemic alterations across the epigenetic landscape. The epigenetic remodelling triggered helps to re‐establish the functional and structural balance of the affected genomes (e.g. Song & Chen, 2015; Alonso *et al.*, 2016), but it can have ecological implications (e.g. Paun *et al.*, 2010). Given their prevalence, disentangling the effects of hybridization and polyploidy from one another promises to improve our knowledge about plant genome evolution. Li *et al. *(2019) report a large‐scale methylome stability across several rice accessions, including diploid parents, diploid F_1_ hybrids and allotetraploids. However, they observed in the allopolyploids considerable but regional re‐patterning in the DNA methylation landscape that was missing in the diploid hybrid genomes, indicating that in this system hybridity can exacerbate, only in combination with polyploidy, a rewiring of epigenetic and gene expression landscape. The instability in the DNA methylation landscape is even greater in the case of interploidal hybrids as shown in a study of several natural and artificial cytotypes of *Solanum* (Cara *et al.*, 2019).

The remodelling of the epigenetic landscape can be seen as a consequence of a phenomenon long depicted as ‘genomic shock’ (McClintock, 1984). The strength and effect of this ‘stress state’ is determined by the divergence in TE load between the parental species (Mhiri *et al.*, 2018). These ‘selfish’ and often deleterious elements can be seen as genomic magnets for epigenetic silencing. Building on these ideas, it has been proposed that the subgenome with a higher TE load will have more genes in proximity to TEs, therefore at greater silencing risk, causing an expression bias towards the genome with less TE load (Woodhouse *et al.*, 2014; Gaebelein *et al.*, 2019). Similarly, it has been shown that the dominant genome can be targeted by less silencing in comparison to the parental level, while the recessive genome retains the parental level of silencing (reviewed in Bird *et al.*, 2018). The ‘TE model’ of subgenome‐specific bias and its influence on *cis–trans* interactions and homoeologue expression are discussed in a recent paper by Hu & Wendel (2019). In addition to the ‘TE model’, a transcription factor model is also highlighted as a strong candidate in shaping the fate of the divergent subgenomes (Hu & Wendel, 2019).

## Epigenetics and plant development

Epigenetic mechanisms are also major players during plant development, and have a role in shaping phenotypic plasticity (Gallusci *et al.*, 2017). The latest advances in understanding the epigenetic dynamics associated with reproductive development in angiosperms are reviewed by Gehring (2019). Gehring stresses that epigenetic reprogramming during plant reproductive development does not entail a genome‐wide erasure of epigenetic signals, in stark contrast for example to mammals. A particular dynamism during reproductive development has been observed in DNA methylation in the CHH context (Gehring, 2019), but their relevance is still unclear and a topic for forthcoming studies.

Histone modifications also appear involved in fine‐tuning different phases of plant development. They can, for example, influence flowering time (Crevillen *et al.*, 2019) and the switch from the heterotrophic to the photosynthetic stages during early seedling development in *Arabidopsis* (Benoit *et al.*, 2019). Through the use of mutants and crossing experiments a further study by Zhao* et al*. (2019) uncovers interactive roles of the HUB2 and SDG8 histone‐modifying enzymes in controlling expression of specific genes, regulating proper plant growth and development. The dynamics of chromosomal states during development is linked to the activity of a histone chaperone, chromatin assembly factor 1 (CAF‐1; Cheloufi *et al.*, 2015). Starting from the observation that selfing CAF‐1 mutants over generations progressively aggravates the specific phenotype, Mozgova *et al*. (2018) show that this is linked to an increasing upregulation of plant defence‐related genes that has an epigenetic nature. Through crossings of different generations of selfed mutants they also document a preferred maternal transgenerational transmission of the phenotype.

Finally, epigenetic mechanisms involving small RNAs, in particular siRNAs, are relevant during plant reproduction, for example during a phase of global reactivation of TEs in gametes. During this stage an intercellular movement of siRNAs between companion cells and male gametic cells has been observed, and recent studies have elucidated the function of sperm‐delivered siRNAs during early seed development (reviewed by Wu & Zheng, 2019).

‘Our understanding of the control and function of structural modifications to DNA has, in recent years, been complemented by developmental and ecological perspectives of epigenetics.’

## Conclusions

Our understanding of the control and function of structural modifications to DNA (e.g. Law & Jacobsen, 2010) has, in recent years, been complemented by developmental and ecological perspectives of epigenetics (Richards *et al.*, 2017; Alonso *et al.*, 2019a; Gáspár *et al.*, 2019). Important examples are described in detail in the present Virtual Issue. Several review articles, including Tansley insight papers in this issue define current research topics and set the foundation for forthcoming themes in plant epigenetics. Given the recent conceptual advances (e.g. Douma *et al.*, 2017; Alonso *et al.*, 2019b; Johannes & Schmitz, 2019), the relevant methodological developments (e.g. Paun *et al.*, 2019), and the rapid increase of available genomic resources for a broad array of organisms, there is no doubt that plant epigenetics will continue to thrive and deliver important scientific insights, building on the foundation set by previous research.
